# Molecular cloning and expression analysis of the Synaptotagmin-1 gene in the hypothalamus and pituitary of Huoyan goose during different stages of the egg-laying cycle

**DOI:** 10.1186/1477-7827-12-83

**Published:** 2014-08-21

**Authors:** Xinhong Luan, Lina Luo, Zhongzan Cao, Rongrong Li, Dawei Liu, Ming Gao, Mei Liu, Laiyou Wang

**Affiliations:** College of Animal Science and Veterinary Medicine, Shenyang Agricultural University, Shenyang, 110866 China; Liaoning Province Livestock and Poultry Genetic Resources Conservation and Utilization Center, Liaoyang, 111000 China

**Keywords:** Huoyan goose, Syt1, Hypothalamus, Pituitary, cDNA, RACE, Real time RT-PCR

## Abstract

**Background:**

Synaptotagmin-1 (Syt1) is an abundant, evolutionarily conserved integral membrane protein that plays essential roles in neurotransmitter release and hormone secretion. Neurotransmitters secreted by hypothalamic neurons can alter GnRH (gonadotropin-releasing hormones) neuronal activity by binding to and activating specific membrane receptors in pituitary cells and, in turn, control the release of gonadotropin hormones from the pituitary gland. To reveal the influence of Syt1 on the process of goose egg-laying, we cloned and characterized the cDNA of goose Syt1 originating from hypothalamus and pituitary tissues of Huoyan goose and investigated the mRNA expression profiles during different stages of the egg-laying cycle.

**Methods:**

Hypothalamus and pituitary tissues were obtained from 36 Huoyan geese in the pre-laying period, early laying period, peak-laying period, and ceased period. The cDNA sequences of goose Syt1 were cloned and characterized from Huoyan goose tissues using 5’-RACE and 3’-RACE methods. Multiple alignments and phylogenetic analyses of the deduced Syt1 amino acid sequence were conducted using bioinformatics tools. The expression profiles of the Syt1 mRNA in the hypothalamus and pituitary during pre-laying, early laying, peak-laying and ceased period were examined using real-time PCR (qRT-PCR).

**Results:**

The cDNA of Syt1 consisted of a 274 bp 5’ UTR, a 1266 bp open reading frame (ORF) encoding 421 amino acids, and a 519 bp 3’ UTR. The deduced amino acid sequence of goose Syt1 is highly conserved with the sequence from other species, especially with birds (more than 98%), and contains two protein kinase C2 conserved regions (C2 domain) from amino acids residue 157 to 259 and 288 to 402. The results of qRT-PCR demonstrated that the expression of Syt1 mRNA increased from the pre-laying period to the peak-laying period, reached its peak in the peak-laying period, and then decreased in the ceased period.

**Conclusions:**

To the best of our knowledge, this study is the first to obtain full-length cDNA sequences of the goose Syt1 gene, and the results of Syt1 mRNA expression profiling in the hypothalamus and pituitary tissues suggested that Syt1 may play an important role in regulating the secretion of hormones relevant to the reproduction and egg-laying of female geese.

## Background

Synaptotagmins (Syts) are abundant, evolutionarily conserved integral membrane proteins that play essential roles in neurotransmitter release and hormone secretion, and regulate exocytosis in nervous and endocrine systems. They contain a short intraluminal N-terminal region, a single transmembrane domain, and two cytoplasmic PKC-homologous repeats (C2A and C2B domains) that bind Ca^2+^ via negatively charged aspartate residues [1]. Syts have been grouped into three classes: A, B and C, based upon their (C2A-domain) calcium dependent binding of syntaxin. Synaptotagmin-1 (Syt1) belongs to class A, is involved in the secretions from synaptic vesicles at synapses and is the most widely distributed synaptotagmin isoform in the nervous and endocrine systems [2]. In particular, it has been reported to be expressed in the hypothalamus and pituitary [3].

The roles of Syt1 have been extensively studied for neurotransmitter and hormone release. Neurotransmitter and hormone releases are triggered by Ca^2+^ binding to a presynaptic Ca^2+^ sensor that induces synaptic vesicle exocytosis with a high degree of Ca^2+^ cooperativity. Syt1 has been identified as a primary Ca^2+^ sensor in synaptic vesicle exocytosis and is a major transducer of Ca^2+^ signaling in membrane fusion events and regulated secretion [4–6]. There is evidence that Syt1 plays a physiological role in secretion by differentiated pituitary cells. Genetic experiments in mice have demonstrated that Syt1 mutants cause defects in regulating secretions [4]. Mice with homozygous disruption of the Syt1 die shortly after birth and have defects in neurotransmitter release from hippocampal neurons [7]. The pituitary-specific transcription factor (POU1F1) is a factor that binds to and activates growth hormone (GH) promoters and is important for the proper development of the pituitary cells that express GH and thyroid stimulating hormone (TSH). There is evidence that it can bind to a specific site in the Syt1 gene and this binding contributes to the activation of Syt1 expression. It appears likely that activation of Syt1 gene expression is part of a mechanism mediating POU1F-induced differentiation of pituitary cells and presumably contributes to the endocrine/secretory phenotype [8].

In poultry, the reproductive endocrine system and reproductive activity are strictly controlled by the hypothalamic-pituitary-gonadal axis [9]. The hypothalamus regulates reproduction by releasing neurohormones (gonadotropin-releasing hormones, GnRH) to the pituitary gland, and the pituitary gland synthesizes and releases gonadotropins (luteinizing hormone, LH; follicle-stimulating hormone, FSH) which, in turn, act on the gonads to stimulate gametogenesis (spermatogenesis, oogenesis) and sex steroid hormone secretion (androgens, estrogens, and progesterone). It is clear that regulation of the synthesis and secretion of GnRH, LH and other hormones is affected by neurotransmitter systems in the hypothalamus and pituitary [10]. Changes in neurotransmitter output and, in particular, alterations in the secretion of monoamines, dopamine, glutamate, noradrenaline, and serotonin have been associated with hormonal changes in mammals [11–14]. The effect of neurotransmitters on the release of pituitary hormones in birds has also been investigated. Monoamines and dopamine were confirmed to affect pituitary release of prolactin (PRL) and GH in the pituitary-hypothalamus of avian species [15, 16]. Our previous research showed that Syt1 was up-regulated in the pituitary gland of laying-period Huoyan geese compared with those of ceased period geese [17]. Therefore, we hypothesize that Syt1 may play an important role in regulating secretion of hormones and the reproductive functions of the female goose. However, to our knowledge, the molecular characterization of the Syt1 gene in geese has not yet been reported, and the expression profiling of Syt1 in the hypothalamus and pituitary of geese during different stages of the egg-laying cycle remains to be determined.

In this study, the full-length cDNA of Syt1 of the Huoyan goose was obtained by RACE (rapid amplification of cDNA ends), and the sequence of Syt1 was analyzed. The expression profiles of the Syt1 mRNA in the hypothalamus and pituitary during pre-laying, early laying, peak-laying and ceased period were examined using real-time PCR (qRT-PCR).

## Methods

### Animal and tissue collection

This study was reviewed and approved by the Institutional Animal Care and Use Committee of the College of Animal Science and Veterinary Medicine of Shenyang Agricultural University and performed in accordance with the Regulations for the Administration of Affairs Concerning Experimental Animals (China, 1988) and the EU Directive 2010/63/EU for animal experiments. Thirty-six Huoyan geese were selected randomly from two hundred geese on the Liaoning Huoyan goose stock breeding farm and were reared according to the program used at the farm. During the experiment, geese were fed rice grain ad libitum, which was supplemented with green grass or water plants whenever possible. Feeding occurred during the daytime when the geese were released into an open area outside of the house. Huoyan geese become sexually mature at approximately 7 months of age and reach the peak egg-laying stage in the following year. In the current study, goslings were purchased in the fall of the year and become sexually mature during the summer of the following year. Nine geese were exsanguinated during each period: 6 months of age (pre-laying period), 9 months of age (early laying period), 12 months of age (peak-laying period), and 15 months of age (ceased period). The hypothalamus and pituitary were quickly dissected, frozen in liquid nitrogen, and stored at −80°C until total RNA extraction.

### RNA isolation and amplification of cDNA

Total RNA was extracted using Trizol reagent (Invitrogen Corporation, Carlsbad, CA) following the manufacturer’s protocol. The quality of the RNA was determined using agarose gel electrophoresis and a NanoDrop 8000 spectrophotometer (NanoDrop, Thermo Scientific). One microgram of RNA was reverse transcribed into cDNA using a PrimeScript®RT reagent Kit (TaKaRa, Dalian, China) in a 20 μl reaction volume containing 4.0 μl of 5 × PrimeScript®Buffer, 1.0 μl of PrimeScript®RT Enzyme Mix, 2.0 μl of oligo(dT)_18_ Primer, and the final volume was adjusted using RNase-free water. Thermal cycling was performed for 15 min at 37°C, then 5 s at 85°C. RT products were stored at 20°C for the RT-PCR.

According to the mRNA sequence of the Gallus gallus Syt1 gene (Genbank accession no. NM_205171.1), a pair of primers (Syt1-F/Syt1-R) was designed to obtain a partial goose Syt1 gene sequence (primers shown in Table 1) by using Primer Premier 6.0 software (Primer Biosoft International, Palo Alto, California, USA). The primer pairs were synthesized commercially by Sangon Biotech Co., LTD (Shanghai, China). The 50 μl reaction consisted of 1 μl of cDNA, 8 μl of deoxynucleoside triphosphate mix (2.5 mmol/L each dATP, dGTP, dCTP and dTTP), 2 μl of each primer (10 μmol/l), 5 μl of 10 × LA PCR Buffer, 0.5 μl of 5U/μl LA Taq™ (TaKaRa, Dalian, China), and 31.5 μl sterile MilliQ water. The PCR program include denaturation at 94°C for 5 min, followed by 35 cycles of 30 s at 94°C, 30 s at 60°C, 60 s at 72°C, and an extension step of 10 min at 72°C. The PCR products were gel-purified and ligated into pMD-18-T vector (TaKaRa, Dalian, China), transformed into the E. coli DH5α competent cell. Positive clones containing the expected-size inserts were screened with colony PCR and then sequenced by Sangon Biotech Co., LTD.

**Table 1:** **Primers used in this study**

Primers purpose	Primer name	Primer sequence (5′-3’)
RT-PCR	Syt1-F	AACCCTGTTTTCAATGAGCAA
	Syt1-R	CACTATGTGGGCAAATGCAG
3’-RACE	Syt1-GSP3	GGGCTACAACAGCACTGGAGCGGAG
	Syt1-NGSP3	CTTGCAGCCCGAGGAGGAGGTAGAT
5’-RACE	Syt1-GSP5	CCGTGTTCATCGCAACCTTA
	Syt1-NGSP5	TTCCACCCAGCTCGGAGTAT
RACE	UPM-Long	CTAATACGACTCACTATAGGGCAAGCAGTGGTATCAACGCAGAGT
	UPM-Short	CTAATACGACTCACTATAGGGC
Real-time PCR	Syt1-S	TATGACAAGATTGGCAAGAAC
	Syt1-A	GGCATCTACCTCCTCCTC
Internal control	18S rRNA-S	CGGACAGGATTGACAGATTGAG
	18S rRNA-A	GCCAGAGTCTCGTTCGTTAT

Based on the partial goose Syt1 cDNA sequence obtained from the above RT-PCR reaction, goose gene specific primers were designed to amplify the full-length cDNA sequence of goose Syt1 (primers shown in Table 1) using the SMARTer™ RACE cDNA Amplification kit (Clontech Laboratories, CA, USA) according to the manufacturer’s instructions. The 3’- and 5’-end cDNA templates were synthesized using the 3’-CDS Primer A and 5’-CDS Primer A provided in the kit. Nested PCR was used in the 3’-RACE analysis. The first-round PCR was performed in a total volume of 50 μl that contained 2.5 μl of the first strand 3’- end cDNA template, 5.0 μl of 10× Advantage 2 PCR buffer, 1.0 μl of 10 mM dNTP Mix, 1.0 μl of 10 μM gene-specific primer Syt1-GSP3, 5.0 μl of 10 × Universal Primer Mix (UPM; Clontech, USA), 34.5 μl of sterile deionized water, and 1.0 μl of 50 × Advantage 2 Polymerase Mix (Clontech, USA). Then, 1 μl PCR product was diluted to 1:50 and subsequently amplified with the Syt1-NGSP3 and UPM as described above. For the 5’ RACE, a 5’- end cDNA template, SMARTer™ cDNA kit UPM and the gene-specific primer Syt1-GSP5 were used for the first-round PCR. These amplified products were then subjected to a second round of nested PCR with the UPM and Syt1-NGSP5. PCR amplification conditions for 3’ and 5’ RACE were as follows: 5 cycles at 94°C for 30 s and 72°C for 3 min; 5 cycles at 94°C for 30 s, 70°C for 30 s, and 72°C for 3 min; 25 cycles at 94°C for 30 s, 68°C for 30 s, and 72°C for 3 min; a final extension for 10 min at 72°C; and then cooled to 4°C.

### Cloning and sequencing

The final PCR products were gel-purified and ligated into pMD-18-T vector (TaKaRa, Dalian, China) and then transformed into the E. coli DH5α competent cell. Positive clones containing the expected-size inserts were screened using colony PCR and then sequenced by Sangon Biotech Co., LTD.

### Bioinformatic analysis

The data of DNA sequences were edited and analyzed using Lasergene 7.0 software (DNA Star Inc., Madison, USA), and similarity analyses of nucleotide and protein sequences were carried out using the BLAST program from the NCBI (http://blast.ncbi.nlm.nih.gov/Blast.cgi). The open reading frame (ORF) was obtained using the ORF finder (http://www.ncbi.nlm.nih.gov/gorf/gorf.html), and the coding region sequences were translated into amino acid sequences using the sequence manipulation suite (SMS) tool (http://www.bio-soft.net/sms/index.html). The homologous conserved domains were identified with SMART (Simple Modular Architecture Research Tool, http://smart.embl-heidelberg.de). The molecular weight and isoelectric point of this predicted protein were analyzed using the ExPASy ProtParam tool (http://www.expasy.org/tools/protparam.html). The PSORT II web-based program (http://psort.hgc.jp/form2.html) was used to predict the subcellular distribution of the Syt1 protein. The presence of transmembrane regions, phosphorylation sites, N-glycosylation sites and the secondary structure of the Syt1 protein were predicted using the TMHMM, version 2.0; NetPhos, version 2.0; NetNGlyc, version 1.0; and SOPMA web-based programs, respectively. Multiple alignments of the Syt1 sequences were performed with the ClustalX2 program [18] and the phylogenetic tree was constructed using the neighbor-joining (NJ) methods (bootstrap phylogeny test, 1000 replicates) with the MEGA 4.0 program [19].

### Quantitative real-time RT-PCR analysis

To evaluate the gene expression changes of Syt1 in hypothalamus and pituitary tissues of Huoyan geese during different stages of the egg-laying cycle, qRT-PCR was performed. The primers used in qRT-PCR are listed in Table 1. Total RNA was extracted using trizol reagent (Invitrogen Corporation, Carlsbad, CA) according to the manufacturer’s instructions. The concentration and purity of the RNA were measured as described above. Two micrograms of total RNA was reverse transcribed using a PrimerScript® RT reagent Kit (TaKaRa, Dalian, China). Real-time PCR was carried out on the Bio-Rad iQ5 Real-time PCR Detection System (BIO-RAD, California, USA). Each 25 μl reaction volume contained 1 μl 10 μM (each) forward and reverse primers, 12.5 μl 2 × SYBR® Premix Ex Taq™ II (Takara, Dalian, China), and 2 μl cDNA products, and the final volume was adjusted using PCR-water. The following PCR program was used for amplification: 5 min at 95°C, 40 cycles of denaturation at 95°C for 10 s and annealing and extension at 60°C for 30 s. Then, 18S rRNA was selected as an internal reference gene and the expression level was used to normalize the qRT-PCR results. Negative controls without the cDNA template were included in this experiment. The standard curve testing was performed using a series of 10-fold diluted samples. The slopes of standard curves and PCR efficiency were calculated to determine whether the qRT-PCR data were precise and trustworthy. Melting curves were analyzed to ensure that a single PCR product was amplified for each pair of primers. Product purity was confirmed with electrophoresis. All samples were amplified in triplicate.

### Statistical analysis

Threshold and Ct (threshold cycle) values were determined automatically by the Bio-Rad iQ5 Real-time PCR Detection software using default parameters. The relative levels of expression for Syt1 were calculated relative to 18S rRNA using the 2^−ΔΔCt^ method [20]. The mRNA level of Syt1 in the pre-laying period was assigned a value of 1. All data were performed using SPSS 16.0 for Windows (SPSS Inc. Chicago, Illinois, USA). The data were analyzed with one-way ANOVA, followed by Tamhane’s T2 post hoc test. The results are expressed as the mean ± SEM. *P* < 0.05 was considered statistically significant.

## Results

### Cloning and characteristics of the Syt1 cDNA

**Figure 1:**
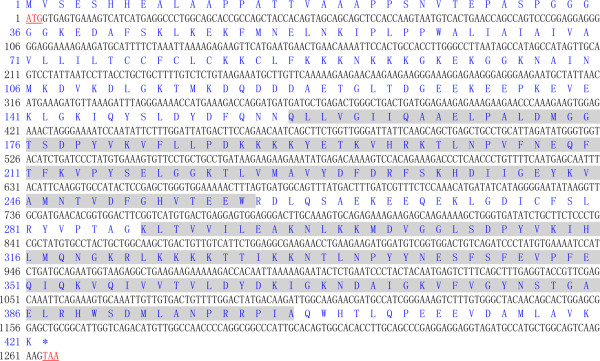
**Nucleotide and deduced amino acid sequences of Syt1.** The nucleotide (black) and deduced amino acid (blue) sequences are shown and numbered on the left. The nucleotide sequence is numbered from the 5’ end. The first methionine (M) is the first deduced amino acid. Two protein kinase C2 conserved regions (amino acids 157–259, 288–402) are shaded. The start codons (ATG) and the stop codons (TAA) are marked in bold red.

The full-length cDNA of the goose Syt1 gene was synthesized as described above. It is 2059 bp in length (Genbank accession no: KJ734994) and consists of a 274 bp 5’ UTR, a 1266 bp ORF encoding 421 amino acids (Figure 1), and a 519 bp 3’ UTR. According to the prediction of the ProtParam, the molecular mass of the goose Syt1 protein is 47.207 kDa, and the theoretical isoelectric point is 8.43. Under the analysis of the deduced amino acid sequence by the SMART program, Syt1 contained two protein kinase C conserved regions (C2 domain) from amino acid residues 157 to 259 and 288 to 402. The subcellular distribution of the Syt1 protein was predicted to be 43.5% in cytoplasm, 26.1% in mitochondria, 8.7% in nuclear, 8.7% in endoplasmic reticulum, 4.3% in vesicles of secretory system, 4.3% in Golgi and 4.3% in peroxisomal. One transmembrane domain was found from amino acids residue 60 to 82. Twenty putative phosphorylation sites were identified in the Syt1 protein, which included six serine residues (Ser5, Ser31, Ser43, Ser217, Ser235, and Ser344), seven threonine residue (Thr17, Thr115, Thr128, Thr176, Thr201, Thr211, and Thr329), and seven tyrosine residues (Tyr151, Tyr180, Tyr193, Tyr216, Tyr282, Tyr311, and Tyr364). Two putative 197 N-glycosylation sites were identified located in amino acid positions 25 and 381. The secondary structure of the Syt1 protein was predicted to consist of 33.25% α-helix, 19% extended strand, 2.61% β-turn, and 45.13% random coil.

### Sequence alignment and phylogenetic analysis

**Figure 2:**
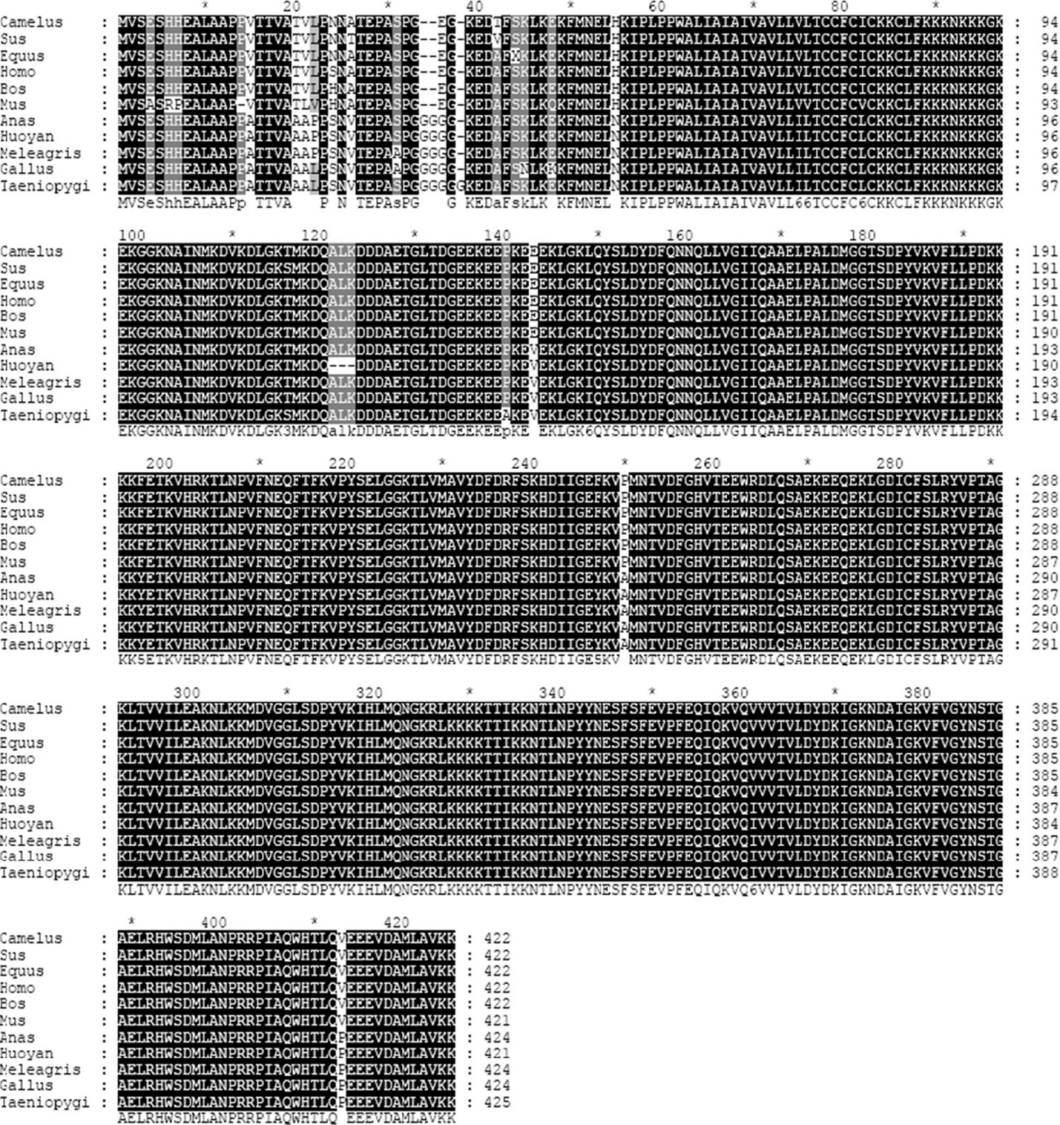
**Multiple amino acid sequence alignment of the Huoyan goose Syt1 protein with other vertebrate species.** The sequences were compared by ClustalX2 Multiple Sequence Alignment Program software. The color black denotes 100% conserved sequences, and the color gray indicates non-conservative sequences. Gaps (−) were introduced to maximize the alignment. Sequences for the alignment were obtained from GenBank (accession numbers are in brackets): *Anas platyrhynchos* (EOB07740.1); *Bos taurus* (NP_776617.1); *Camelus ferus* (XP_006179157.1); *Equus caballus* (XP_005606551.1); *Gallus gallus* (NP_990502.1); *Homo sapiens* (NP_001129278.1); *Meleagris gallopavo* (XP_003202150.1); *Mus musculus* (NP_001239270.1); *Sus scrofa* (XP_005652679.1); and *Taeniopygia guttata* (NP_001041725.1).

The amino acid sequence identities of the Huoyan goose Syt1 with the other representative species were investigated via multiple sequence alignment on the ClustalX2 program (Figure 2). The overall percent identity among these Syt1 sequences is shown in Table 2.

**Figure 3:**
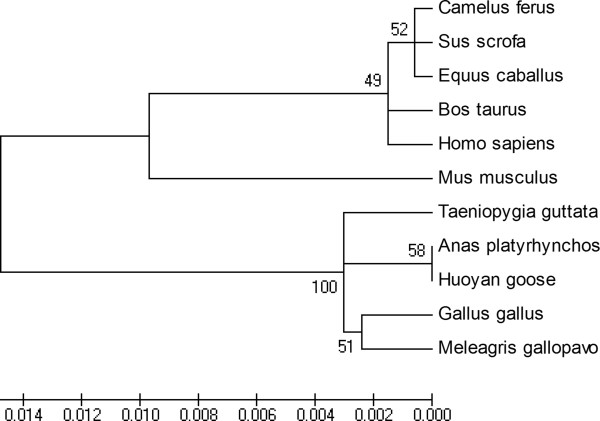
**Phylogenetic tree of Syt1.** The phylogenetic tree of Syt1 protein was constructed using the neighbor-joining method with MEGA4. Amino acid sequences of Syt1 for these species were downloaded from the protein database of the NCBI. Their corresponding accession numbers are the same as those given in Table 2. The number at the branches denotes the bootstrap majority consensus values on 1000 replicates; the branch lengths represent the relative genetic distances among these species.

A phylogenetic tree was constructed using the MEGA program based on the amino acid sequences of the Huoyan goose Syt1 and the other species previously mentioned (Figure 3). It was clustered into two subgroups, the avian species (including goose, duck, turkey, chicken and zebra finch) belonging to one group, and the mammalian species belonging to another one. The phylogenetic tree indicated that the deduced goose Syt1 protein showed a closer genetic relationship to the avian species Syt1 than to those of the mammal species.

**Table 2:** **Syt1 amino acid sequence identities between the Huoyan goose and ten other vertebrate species**

Matched species	GenBank accession no.	% Identity
Duck (*Anas platyrhynchos*)	EOB07740.1	99
Cattle (*Bos taurus*)	NP_776617.1	95
Camel (*Camelus ferus*)	XP_006179157.1	95
Horse (*Equus caballus*)	XP_005606551.1	95
Chicken (*Gallus gallus*)	NP_990502.1	98
Human (*Homo sapiens*)	NP_001129278.1	95
Turkey (*Meleagris gallopavo*)	XP_003202150.1	99
Mouse (*Mus musculus*)	NP_001239270.1	93
Pig (*Sus scrofa*)	XP_005652679.1	94
Zebra finch (*Taeniopygia guttata*)	NP_001041725.1	98

### mRNA expression of Syt1 mRNA in hypothalamus and pituitary

**Figure 4:**
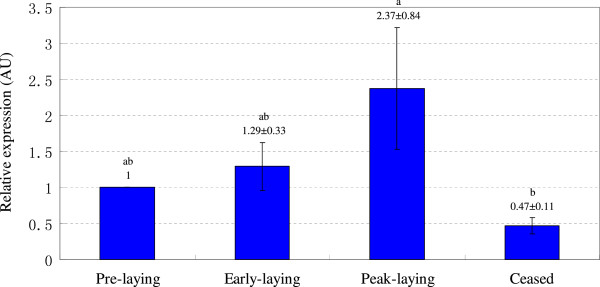
**Relative expression of Syt1 mRNA in the hypothalamus of Huoyan geese during different stages of the egg-laying cycle.** The expression levels of Syt1 were normalized 18S rRNA. The expression levels, calculated by the 2^−ΔΔCt^ method, are presented in arbitrary units (AU). Values are the means ± SEM. The significance of differences in the levels of expression of Syt1 mRNA was determined by ANOVA followed by Tamhane’s T2 test post hoc test. The means marked with the same letter are not significantly different (*P* < 0.05).

**Figure 5:**
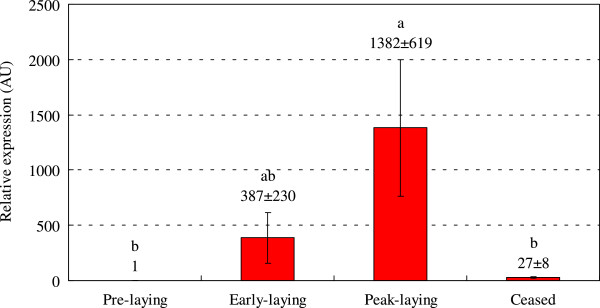
**Relative expression of Syt1 mRNA in the pituitary of Huoyan geese during different stages of the egg-laying cycle.** The expression levels of Syt1 were normalized 18S rRNA. The expression levels, calculated by the 2^−ΔΔCt^ method, are presented in arbitrary units (AU). Values are the means ± SEM. The significance of the differences in the levels of expression of Syt1 mRNA was determined using ANOVA followed by Tamhane’s T2 post hoc test. The means marked with the same letter are not significantly different (*P* < 0.05).

The mRNA levels of Syt1 in the hypothalamus and pituitary of Huoyan geese during pre-laying period, early laying period, peak-laying period, and ceased period were determined with qRT-PCR. As shown in Figure 4, in the hypothalamus, the expression of Syt1 mRNA increased from the pre-laying period to the peak-laying period, reached its peak in the peak-laying period, then decreased and reached its lowest expression in the ceased period. The expression of Syt1 was significantly higher in the peak-laying period compared with ceased period (*P* < 0.05). Similarly, as shown in Figure 5, the expression of Syt1 mRNA in the pituitary increased from the pre-laying period to the peak-laying period, reached its peak in the peak-laying period, and then decreased in ceased period. The expression of Syt1 in the peak-laying period was significantly higher than for the pre-laying period and ceased period (*P* < 0.05).

## Discussion

Syts are a large family of single-pass transmembrane proteins found in diverse populations of intracellular vesicles containing various hormones [21]. Vesicles harboring different synaptotagmin isoforms can preferentially undergo distinct modes of exocytosis with different forms of stimulation, which can shape Ca^2+^ sensing in endocrine cells, contributing to the regulation of hormone release and the organization of complex endocrine functions [1]. Exocytosis is a key biological process that controls the neurotransmission and release of secretory products from neurons and other secretory cell types. Neurotransmitters, hormones, or other secretory products are packed in vesicles, and a number of these vesicles fuse with the surface membrane during both nonactivated and activated phases to release a secretory product into the extracellular space [22]. At present, members of the Syts protein family are the most probable candidates to function as Ca^2+^ sensors during regulated exocytosis.

Syt1 has been extensively studied as a major transducer of Ca^2+^ signaling in membrane fusion events and regulated secretion. In addition to its location on synaptic vesicle membranes, Syt1 protein has also been found on large dense core vesicle membranes of the rat hypothalamus and on granules of the cells in the anterior and intermediate lobes of the rat pituitary [23, 24]. Synaptic vesicle exocytosis involves three consecutive stages: synaptic vesicle docking (“docking”), acquisition of competence of docked synaptic vesicles to undergo Ca^2+^-dependent fusion (“competence acquisition” or “cocking”), and the actual fusion reaction itself (“fusion”) [7]. Syt1 can specifically interact with several synaptic proteins such as syntaxin, neurexins, and the clathrin assembly protein complex AP-2 [25–27]. Based on these interactions, Syt1 is involved in triggering the final stage of the exocytotic reaction, the fusion reaction.

Neurotransmitter release at synapses is regulated by two kinetically distinct Ca^2+^ sensors. A low-affinity Ca^2+^ sensor mediates the rapid synchronous component of transmitter release, whereas a second Ca^2+^ sensor supports a slower asynchronous phase of fusion. Syt1 has emerged as the primary candidate for the low affinity Ca^2+^ sensor triggering synchronous neurotransmitter release [28, 29]. As an abundant and highly conserved synaptic vesicle protein, Syt1 is composed of a short intravesicular sequence, a single transmembrane region, a variable linker sequence, and two conserved C2 domains referred to as the C2A and C2B domains [30]. Both the C2A and the C2B domains bind Ca^2+^; the C2B domain, which exhibits Ca^2+^/phospholipid binding activity, is the major Ca^2+^ sensor for fast synchronous neurotransmitter release [31], and Ca^2+^ binding to the C2A domain is a major regulator of Ca^2+^ binding to the C2B domain and contributes to the overall Ca^2+^ cooperativity of neurotransmitter release [6].

Cells of the anterior pituitary gland are the source of important physiological hormones. The pituitary secretes LH, FSH, and GH, which controls such important bodily functions as growth, reproduction and egg production. Each pituitary cell is under the control of specific releasing hormones (such as GnRH) and neurotransmitters secreted into the pituitary portal circulation by hypothalamic neurons. Most pituitary cells have receptors for small-peptide-releasing factors (such as GnRH), which are synthesized and secreted from discrete groups of cells in the hypothalamus and possess receptors for neurotransmitter substances and neuropeptides such as excitatory amino acids, y-aminobutyric acid (GABA), 5-Hydroxytryptamine (5HT), acetylcholine, Neuropeptide Y (NPY), noradrenaline and dopamine. By binding to and activating specific membrane receptors, these neurotransmitters and neuropeptides alter GnRH neuronal activity. For example, neurotransmission of excitatory amino acids in the brain principally involves glutamate and aspartate. Excitatory amino acids induce a rapid increase in GnRH mRNA and protein expression, and GnRH or LH release [32]. GABA, the major inhibitory neurotransmitter of the brain, plays an important role in the regulation of GnRH secretion. Removal of GABAergic tone on the afternoon of pro-estrus is an important neural signal for the generation of the LH surge [33]. Electrophysiological studies [34, 35] demonstrated that all GnRH neurons in mice can express functional GABA receptors, which underlines the importance of this neurotransmitter in the control of GnRH neurons. NPY can facilitate GnRH release, potentiate the responsiveness of gonadotrophs to GnRH, and participate in the regulation of several physiological functions such as gonadotropin release, sexual behavior, food intake, energy metabolism, and stress responses [36]. These comprehensive studies demonstrated that regulation of both synthesis and the secretion of GnRH are effected by neurotransmitter systems in the brain. In addition to influencing the release of GnRH, some neurotransmitters, such as dopamine, are also involved in the control of PRL secretion. Drugs that decrease the secretion of dopamine have been found to increase the secretion of PRL, and conversely, drugs that increase the secretion of dopamine reduce the secretion of PRL [37]. In vitro, dopamine inhibited the release of PRL directly from the mammalian pituitary gland [38]. Syt1 is the dominant isoform in both peptide secreting systems (e.g., in pituitary tissues) and in neurotransmitter secreting systems (e.g., in the cerebellum) [3]. As a major Ca^2+^ sensor for dense-core vesicle exocytosis in neuroendocrine cells, Syt1 is up-regulated in parallel with synaptogenesis in the mouse brain [39]. Concerning the roles of Syt1 in neurotransmitter and hormone release, we studied the expression profiles of Syt1 mRNA in the hypothalamus and pituitary of Huoyan geese, sites where neurotransmitters and peptides are abundantly exocytosed via calcium-mediated secretion mechanisms [40]. The expression of Syt1 mRNA increases from the pre-laying period to the peak-laying period and then decreases in the ceased period. Notably, due to the important roles of the pituitary gland in gonadotropin hormone secretion, the mRNA expression in pituitary tissue at the peak-laying period was significantly higher than the pre-laying and ceased periods. The up-regulation of Syt1 might imply enhanced synaptogenesis during the laying period and it may markedly elevate FSH, LH and other sex steroid hormone secretions during this period.

## Conclusions

Our study was the first to demonstrate the presence of Syt1 mRNA in the hypothalamus and pituitary tissues of the Huoyan goose and to analyze the effect of different stages of the egg-laying cycle on the expression of Syt1. Our data support the hypothesis that Syt1 may play an important role in regulating the secretion of hormones relevant to the reproduction and egg-laying of female geese.

## Abbreviations

aa: Amino acid(s)
bp: Base pair(s)
cDNA: DNA complementary to RNA
kDa: Kilodalton
mRNA: Messenger RNA
ORF: Open reading frame
PCR: Polymerase chain reaction
PKC: Protein kinase C
RACE: Rapid-amplification of cDNA ends
rRNA: Ribosomal RNA
UTR: Untranslated Regions.
